# Functional Digestive Symptoms and Quality of Life in Patients with Ehlers-Danlos Syndromes: Results of a National Cohort Study on 134 Patients

**DOI:** 10.1371/journal.pone.0080321

**Published:** 2013-11-22

**Authors:** Jean-David Zeitoun, Jérémie H. Lefèvre, Vincent de Parades, César Séjourné, Iradj Sobhani, Benoît Coffin, Claude Hamonet

**Affiliations:** 1 Department of Gastroenterology and Nutrition, Saint-Antoine Hospital, APHP, Paris, France; 2 Department of Proctology, Deaconesses Hospital, Paris, France; 3 Department of Digestive and General Surgery, Saint-Antoine Hospital, APHP, Paris, France; 4 University Paris VI, Paris, France; 5 Department of Proctology, Saint-Joseph Hospital, Paris, France; 6 Private Office, Marolles en Hurepoix, France; 7 Department of Gastroenterology, Henri Mondor Hospital, APHP, Créteil, France; 8 Department of Gastroenterology, Louis Mourier Hospital, APHP, Colombes, France; 9 University Denis Diderot-Paris VII, Paris, France; 10 Department of Physical and Rehabilitation Medicine, Hôtel Dieu Hospital, APHP, Paris, France; 11 Department of Medicine, University Paris-East-Créteil (UPEC), France; Duke University Medical Center, United States of America

## Abstract

**Background and Objectives:**

Ehlers-Danlos syndromes (EDS) are a heterogeneous group of heritable connective tissue disorders. Gastrointestinal manifestations in EDS have been described but their frequency, nature and impact are poorly known. We aimed to assess digestive features in a national cohort of EDS patients.

**Methods:**

A questionnaire has been sent to 212 EDS patients through the French patient support group, all of which had been formally diagnosed according to the Villefranche criteria. The questionnaire included questions about digestive functional symptoms, the GIQLI (Gastrointestinal Quality of Life Index), KESS scoring system and the Rome III criteria.

**Results:**

Overall, 135 patients (64% response rate) completed the questionnaire and 134 were analyzable (123 women; 91%). Mean age and Body Mass Index were respectively 35±14.7 years and 24.3±6.1 kg/m^2^. The most common EDS subtype was hypermobility form (n=108; 80.6%). GIQLI and KESS median values were respectively 63.5 (27-117) and 19 [13.5-22]. Eighty four percent of patients had functional bowel disorders (FBD) according to the Rome III criteria. An irritable bowel syndrome according to the same criteria was observed in 64 patients (48%) and 48 patients (36%) reported functional constipation. A gastro-esophageal reflux disease (GERD) was reported in 90 patients (68.7%), significantly associated with a poorer GIQLI (60.5±16.8 *versus* 75.9±20.3; p<0.0001). GIQLI was also negatively impacted by the presence of an irritable bowel syndrome or functional constipation (p=0.007). There was a significant correlation between FBD and GERD.

**Conclusions:**

Natural frequency of gastrointestinal manifestations in EDS seems higher than previously assessed. FBD and GERD are very common in our study population, the largest ever published until now. Their impact is herein shown to be important. A systematic clinical assessment of digestive features should be recommended in EDS.

## Introduction

Ehlers-Danlos syndromes (EDS) are a heterogeneous group of heritable connective tissue disorders mainly characterized by joint hypermobility, skin hyperextensibility and tissue fragility [1]. It was described at the beginning of the 20^th^ century by two dermatologists, Edvard Ehlers and Henri-Alexandre Danlos [2,3].

In 1997, the Villefranche classification defined the 6 major forms of EDS [4]. The classical type (I), hypermobility type (II) and vascular type (III) are the most frequent clinical presentations while the 3 remaining forms (kyphoscoliosis type, arthrochalasia type, dermatosparaxis type) seem to be very rare (see Table 1). Mutations in type V and type III collagen are respectively involved in classical and vascular EDS while the 3 last forms of EDS are biologically related to the processing of type I collagen. However, genetic background and pathophysiology of EDS remain to be fully elucidated. Numerous articles in the literature have focused on the vascular type because it is associated with the poorer prognosis [5] but it appears that this body of publications is out of proportion compared to the natural prevalence of respective forms. Hypermobility type is probably more frequent but might be very often undiagnosed [6]. Although gastrointestinal manifestations have already been reported in EDS [7], mainly hemorrhage or perforation, little attention has been paid to functional non-life-threatening gastro-intestinal symptoms which could be frequent considering the importance of collagens in the gut. It has to be noted that most studies recruited a small number of patients. The aim of this study was to describe the type and the frequency of gastrointestinal functional disorders and their impact on quality of life in a large cohort of patients with EDS.

**Table 1:** Nosology from the Villefranche Classification [1].

**Ehlers-Danlos Syndrome**	**Inheritance**	**Major diagnostic criteria**
I, classical type	Autosomal dominant	Skin hyperextensibility
		Widened atrophic scars
		Joint hypermobility
II, hypermobility type	Autosomal dominant	Skin involvement
		Generalized joint hypermobility
III, vascular type	Autosomal dominant	Thin, translucent skin
		Arterial/intestinal/uterine fragility or rupture
		Extensive bruising
		Characteristic facial appearance
IV, kyphoscoliosis type	Autosomal recessive	Generalized joint laxity
		Severe muscle hypotonia at birth
		Scoliosis at birth, progressive
		Scleral fragility and rupture of the ocular globe
V, arthrochalasia type	Autosomal dominant	Severe generalized joint hypermobility with recurrent subluxations
		Congenital bilateral hip disclocation
VI, dermatosparaxis type	Autosomal recessive	Severe skin fragility
		Sagging, redundant skin

## Materials and Methods

An original standardized questionnaire was sent by e-mail or Internet from September 2011 to July 2012 to 212 patients affected by EDS through the French patient support group (*Apprivoiser les Syndromes d’Ehlers-Danlos*, ASED, www.ased.fr). All of the patients had been clinically assessed at least once by a single national expert practitioner in EDS (CH) and all were formally diagnosed as having EDS according to Villefranche criteria [1]. Joint hypermobility was assessed using the Beighton Scale [8]. There was no incentive to complete the questionnaire. Patients who did not complete the survey after one month received a reminder once. It was preplanned that patients who would have responded to the questionnaire improperly would not be recalled. The questionnaire included relevant general and demographic characteristics, and questions about digestive symptoms. Gastro-esophageal reflux disease (GERD) was defined as it is usually done in the scientific literature as the occurrence of either heartburn and (or) regurgitations. Most other esophageal and extraesophageal symptoms of GERD were included in the questionnaire according to their listing in a reference paper [9]. Additionally, 3 internationally validated or at least commonly accepted questionnaires were included which were the following: irritable bowel syndrome (IBS) and functional constipation (FC) were defined according to the Rome III criteria. Severity of constipation was determined by using the Kess score. Impact of these symptoms on quality of life was measured by the Gastrointestinal Quality of Life Index [10] (GIQLI). All these 3 questionnaires had been translated in French. For the Rome III criteria and the Kess score, we used the French versions of the French Group of Neurogastroenterology (*Groupe Français de Neurogastroentérologie*, GFNG) which is the official study group of motility and sensitivity, affiliated to the French National Society of Gastroenterology (SNFGE) and the European Society of Neurogastroenterology and Motility (ESNM). For the GIQLI, we used a validated French version of the questionnaire [11]. Both English and French complete versions of the questionnaires can be found in the Appendix. Data were collected in September 2012 by a single person and entered into a dedicated and anonymized database. This study was approved by the French National Commission for Data Protection (*Commission Nationale Informatique et Libertés*). All patients were fully informed according to the French ethics law and gave written consent.

### Statistical analysis

Statistical analysis was performed by using JMP9 (SAS Institute, USA). Data are shown as the prevalence, mean (standard deviation), or median (range). Continuous data were compared by using the Mann-Whitney U test. Comparison of mean values between three groups (IBS, FC, and no IBS or FC) was performed using ANOVA test. All statistical tests were two-sided, with the threshold of significance set at p<0.05. 

## Results

Out of the 212 patients at baseline, 113 patients returned a questionnaire by e-mail and 22 by mail. Overall, 135 returned a completed questionnaire (64% response rate) among which 134 were analyzable. Demographics and clinical characteristics are shown in Table 2. Gastrointestinal manifestations had been starting before other symptoms of EDS in 54 patients (44.6%) and before EDS formal diagnosis in 96 patients (74.4%). First manifestation of EDS was observed before the age of 18 in 94 patients (69.6%).

**Table 2:** Demographics and clinical characteristics.

**Characteristics**	**n (%)**
Age – mean (± SD)	35 (14.7)
Female	123 (91)
Weight – mean (kg ± SD)	66.6 ± 18.9
Height – mean (cm ± SD)	164.9 ± 10.2
Body mass index – mean (± SD)	24.3 ± 6.1
Age at first symptoms of EDS	
Infant (0-2 years)	26 (19.3%)
Childhood 2-10 years)	41 (30.4%)
Adolescence (11-17 years)	27 (20%)
Adult (> 18 years)	29 (21.5%)
Unknown	12 (8.9%)
Tobacco	39 (31.0)
Daily work	39 (32.5)
Classical type	11 (8.2)
Hypermobility type	108 (80.6)
Vascular type	3 (2.2)
Other – Mixed forms	12 (9)

SD = Standard Deviation.

### Upper GI symptoms: Gastro-esophageal reflux disease (GERD) and dyspepsia

Frequencies of different symptoms of GERD or dyspepsia are shown in Table 3. One hundred and seven patients complained from heartburn and/or regurgitations (79.3%). Seventy-two patients (55%) had undergone upper endoscopy, among which 33 of them (45.8%) were declared to be normal or unremarkable. Nineteen patients had a hiatal hernia on endoscopy.

**Table 3:** Frequencies of upper GI symptoms: gastro-esophageal reflux disease (GERD) or dyspepsia.

**Symptoms**	**n (%)**
Heartburn	90 (68.7)
Regurgitations	90 (68.7)
Symptoms worsened by decubitus	82 (62.6)
Chronic cough	47 (36.2)
Laryngitis	75 (56.8)
Erosion of dental enamel	67 (51.5)
Asthma	58 (45)
Dysphagia	82 (62.6)
Epigastric pain	104 (78.8)
Nausea	92 (70.8)
Postprandial fullness	88 (67.2)
Belching	91 (70.5)

### Lower GI symptoms: irritable bowel syndrome and functional constipation

**Figure 1:**
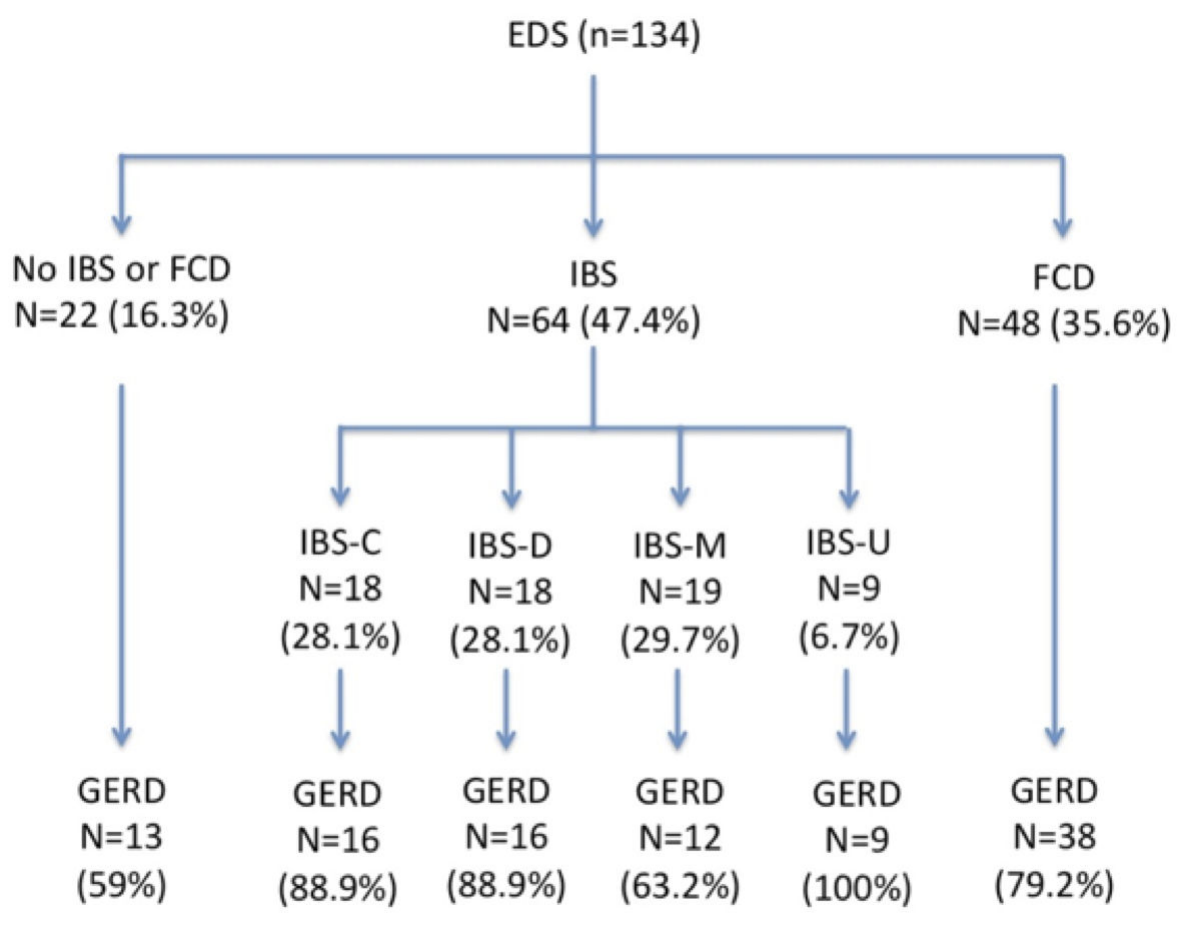
Flow Chart of the functional bowel disorders among the population of Ehlers-Danlos syndrome.

Sixty-four patients (48%) reported irritable bowel syndrome (IBS) according to the Rome III criteria (Table 4) with a homogenous repartition according to stool consistency. Forty-eight patients (36%) displayed functional constipation (FC) according to the Rome III criteria. KESS median score was 19 [13.5-22] and 117 patients (87.3%) had a KESS score > 9, which is the generally accepted cut-off for constipation. Patients with functional constipation had a significant worse KESS score (21.5±0.82 *vs.* 15.8±0.84; p<0.0001). There was a statistically significant association between FBD and GERD: 89.2% (91/102) patients with FBD also had GERD whereas 59.1% (13/22) of patients without FBD were displaying GERD symptoms (p=0.002) (Figure 1). 

**Table 4:** Rome III criteria for Irritable Bowel Syndrome (IBS) and Functional Constipation in the Ehlers-Danlos Syndromes cohort.

**Symptoms**	**N (%)**	**Diagnosis**
**Recurrent abdominal pain or discomfort at least 3 days per month in the last three months associated with:**		
Improvement with defecation	78	
Onset associated with a change in frequency of stool	55	
Onset associated with a change in form (appearance) of stool	66	
**Irritable Bowel Syndrome (IBS) diagnosis**		
3 associated above features	41	64 patients having IBS according to the Rome III criteria
2 associated above features	23	
1 above feature	38	
IBS with constipation		18 (28%)
IBS with diarrhea		18 (28%)
IBS mixed		19 (30%)
Unsubtyped IBS		9 (14%)
**Diagnostic criteria for functional constipation**		
Fewer than 3 defecations per week	37	
Lumpy or hard stools in at least 25% of defecations	64	
Straining during at least 25% of defecations	93	
Sensation of incomplete evacuation for at least 25% of defecations	98	
Sensation of anorectal obstruction/blockage for at least 25% of defecations	54	
Manual maneuvers to facilitate at least 25% of defecations	29	
**Functional Constipation Diagnosis**		
6 associated above features	5	48 patients having functional constipation according to the Rome III criteria
5 associated above features	8	
4 associated above features	6	
3 associated above features	16	
2 associated above features	13	

### Impact on quality of life

**Figure 2:**
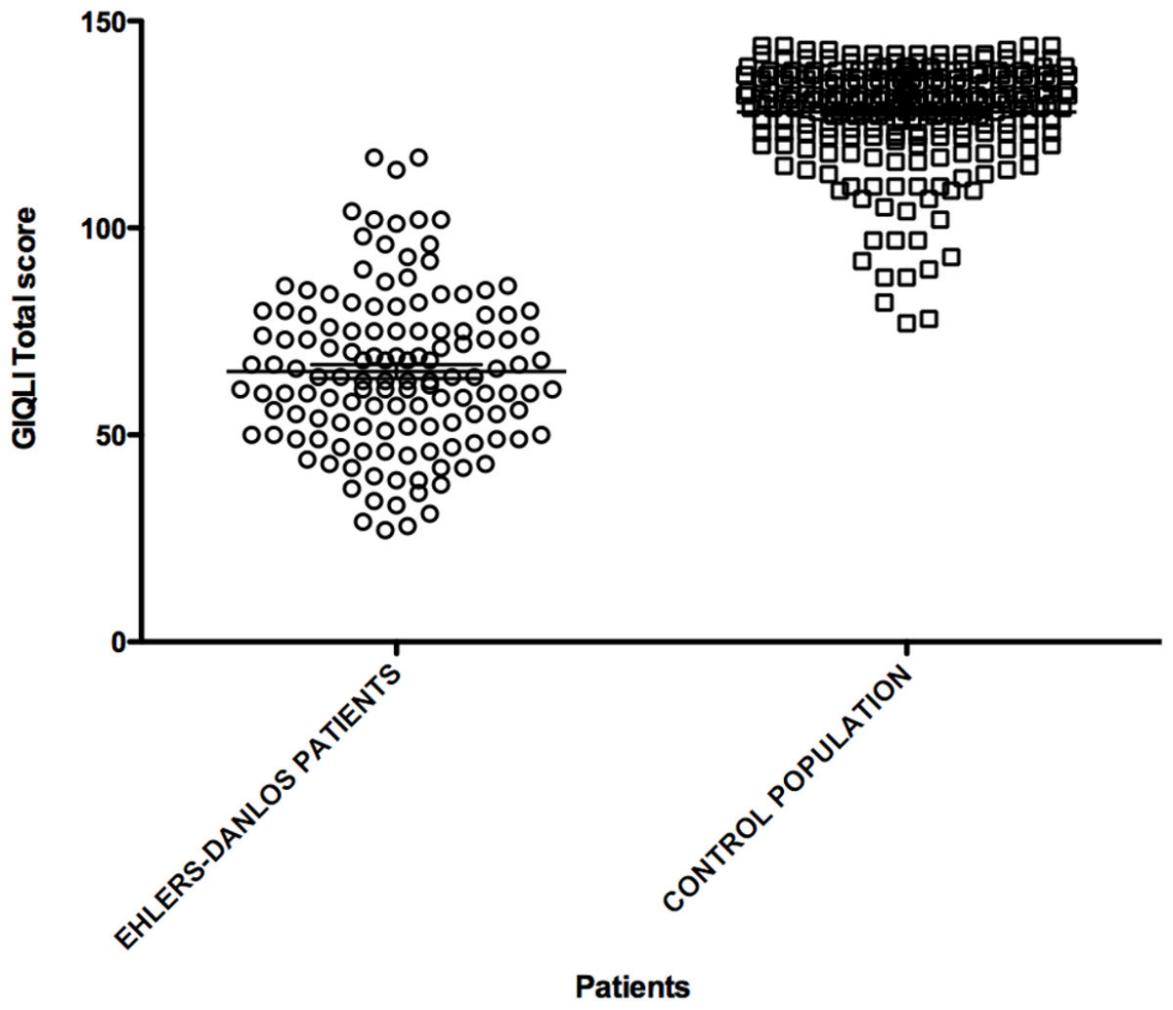
GIQLI score (individual spot and means) according to the presence of Ehlers-Danlos syndrome.

**Figure 3:**
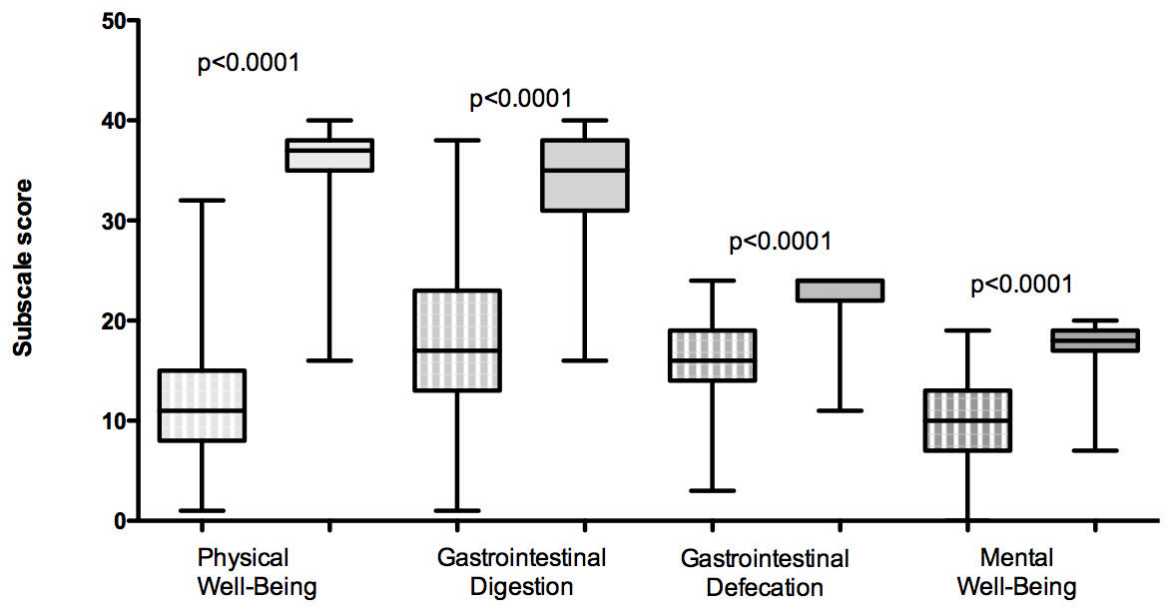
Subscales of the GIQLI between patients with EDS (box with white lines) and control population (filled box) and results of the Student comparison.

Median GIQLI was 63.5 [27-117]. The mean score was significantly lower when compared with a French control population of 238 individuals [11] (65.3±1.65 vs. 128.0±0.81; p<0.0001; see Figure 2). Details and subscales results are presented in Table 5. All subscales scores were significantly lower for patients with EDS when compared to the control groups (p<0.0001), see Figure 3.

**Table 5:** Details of GIQLI score and subscales.

**Items**	Mean±SD	**Subscale score**		
		Theoretical Range	Mean ± SD	Median (min-max)
Physical Well-Being		0-40	11.75±5.59	11(1-32)
Item 8 (Pleasure and appetite)	1.60±1.18			
Item 15 (Fatigue)	0.46±0.71			
Item 16 (Feeling unwell)	1.13±0.92			
Item 18 (Appearance)	1.62±1.47			
Item 19 (Physical strength)	0.82±1.13			
Item 20 (Endurance)	0.35±0.77			
Item 21 (Feeling unfit)	0.40±0.78			
Item 22 (Daily activities)	1.94±1.12			
Item 23 (Leisure activities)	1.44±1.12			
Item 33 (Nausea)	2.08±1.22			
Gastrointestinal digestion		0-40	17.96±7.79	17 (1-38)
Item 1 (Pain in abdomen)	1.65±0.97			
Item 2 (Fullness in abdomen)	1.63±1.27			
Item 3 (Bloating)	1.47±1.17			
Item 4 (Flatus)	1.89±1.28			
Item 5 (Burping/belching)	1.94±1.24			
Item 6 (Abdominal noises)	1.73±1.18			
Item 27 (Regurgitation)	2.01±1.22			
Item 28 (Eating speed)	2.06±1.38			
Item 32 (Constipation)	1.55±1.32			
Item 35 (Heartburn)	2.09±1.28			
Gastrointestinal defecation		0-24	16.01±4.31	16 (3-24)
Item 7 (Bowel frequency)	2.56±1.26			
Item 26 (Impaired sexual life)	1.55±1.26			
Item 30 (Bowel Urgency)	2.44±1.18			
Item 31 (Diarrhoea)	2.82±1.12			
Item 34 (Blood in stool)	3.45±0.94			
Item 36 (uncontrolled stools)	3.47±0.91			
Mental Well Being Scale		0-20	9.80±3.98	10 (0-19)
Item 10 (Coping with stress)	1.90±1.02			
Item 11 (Sad about illness)	2.02±1.07			
Item 12 (Nervous about illness)	2.13±1.20			
Item 13 (Happy with life)	2.23±0.96			
Item 14 (Frustrated by illness)	1.56±0.92			
Items not included in a subscale				
Item 9 (Restricted eating)	2.10±1.38			
Item 17 (Wake up at night)	0.89±1.27			
Item 24 (Bothered by treatments)	2.64±1.19			
Item 25 (Worsened relations)	1.60±1.26			
Item 29 (Dysphagia)	2.61±1.19			
**Overall score**		**0-144**	**65.3±19.1**	**63.5 (27-117)**

SD = Standard Deviation; GIQLI = Gastrointestinal Quality of Life Index.

**Figure 4:**
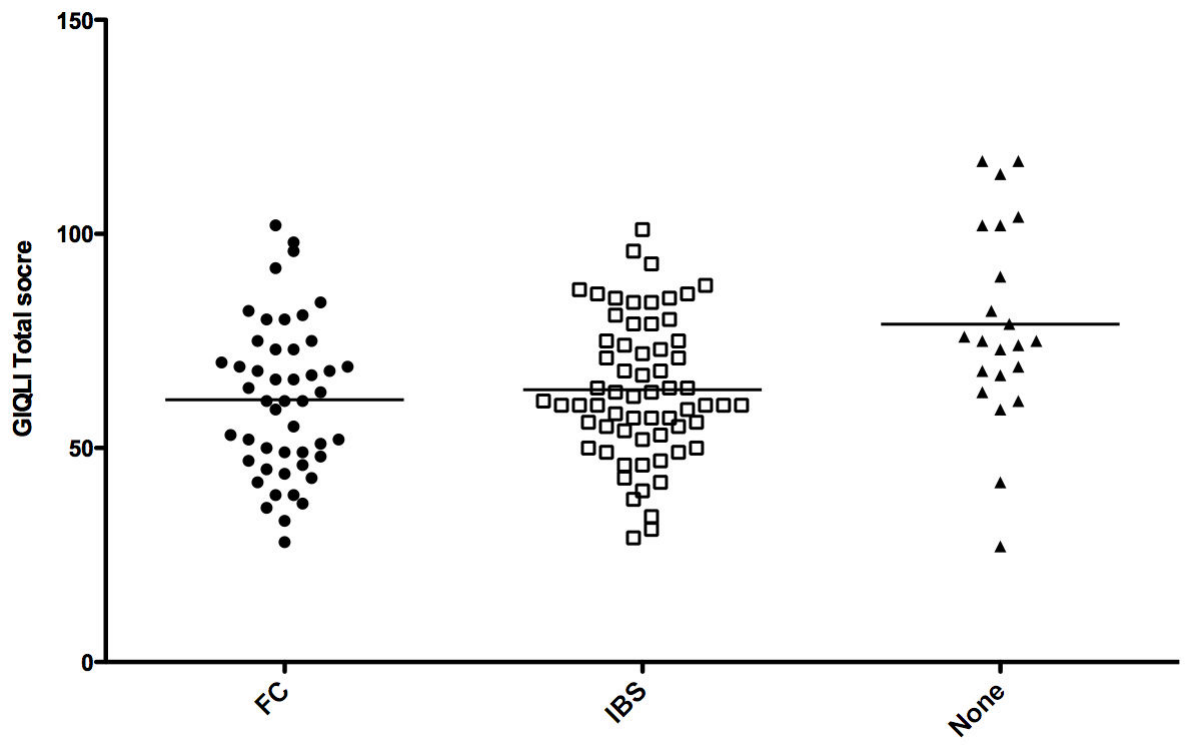
GIQLI score according to the presence and the type of functional bowel disorders (FC: functional constipation; IBS: irritable bowel syndrome).

GERD was significantly associated with a worst GIQLI (60.5 ± 16.8 versus 75.9 ± 20.3; p<0.0001). GIQLI was also significantly lower in case of IBS or FC compared to no FBD (p=0.0007) (Figure 4).

## Discussion

In the present survey, we have shown that the frequency of functional gastrointestinal manifestations in EDS was highly prevalent, much higher than previously assessed. IBS, functional constipation and GERD were present in respectively 48%, 36% and 79% of our study population, the largest ever published until now. Their impact is herein shown to be important. 

Ehlers-Danlos syndromes (EDS) are a genetically and clinically heterogeneous group of disorders characterized by a fragility of the soft connective tissues [12]. The Villefranche classification recognizes six EDS subtypes among which classical, hypermobility and vascular types are the most frequent ones [1]. Most publications concerning gut symptoms in patients with EDS are dealing with vascular type, certainly because it is the most serious one, but gut symptoms occurring in classical or hypermobility types, which affect the majority of patients are paradoxically less documented. No standardized approach has ever been adopted to describe them properly. Only life-threatening complications such as spontaneous perforation or massive bleeding have been reported but one might question the representativeness of such reports regarding the whole population of EDS since it was generally in vascular types of EDS. Our survey is, to our knowledge, the largest in an EDS national cohort assessing gastrointestinal profile of affected patients. All subjects have been formally diagnosed after clinical examination by a single national expert, (CH) according to the validated international Villefranche criteria. Thus, diagnosis of EDS in the respondents of our survey is thought to be reliable and other phenotypically related conditions must have been excluded. Another strength of our study is the use of internationally validated questionnaire and scoring systems to assess clinical features of recruited patients. The major findings of the current study are that gastrointestinal manifestations are very common and generally not specific, frequently important and that they can have a strong impact on quality of life. Eighty four percent of the studied population had FBD among which 57% had IBS and 43% functional constipation. Approximately 80% of patients had GERD. There was a statistically significant association between upper and lower GI symptoms. Overall, median GIQLI was 63.5 [51.8-76.8], which is extremely low compared to most publications and it is of note that GERD and lower GI symptoms negatively influenced this scoring system. All these findings clearly indicate that digestive manifestations in EDS are of major relevance and may have been previously underdiagnosed and undertreated. All reported symptoms are remarkably nonspecific and this could partly explain why little attention has been paid until now to these clinical manifestations. In addition, no severe complication has been described in our population, emphasizing the clear distinction between rare but serious complications of EDS vascular type and other common benign but disabling manifestations, for which literature is scarce. Many questions remain unanswered, among which the pathophysiology of reported symptoms. One might hypothesize that tissue hyperextensibility of the gastrointestinal tract could play a role but proprioceptive disorders as well as dysautonomic syndrome which are very common in EDS [13] could also contribute to gastrointestinal manifestations. In addition, the marked preponderance of affected women *vs.* men in EDS and especially hypermobile type, although previously documented, is still an unexplained feature [6]. Also, current treatment for gastrointestinal symptoms is empirical, often unsatisfactory (data not shown) and further research is needed. At least, a systematic assessment of gastrointestinal symptoms should be recommended in EDS patients in order not to miss a major source of complaints in this population. Whether endoscopic examinations are necessary and at high risk of complication, in particular perforation, is an unresolved question. In our experience, upper gastrointestinal endoscopy seems safe and useful to detect Barrett’s esophagus in this population with a high rate of GERD. On the other hand, the matter of colonoscopy is more sensitive. Indeed, the risk of perforation is clearly significant in vascular EDS and colonoscopy should be strongly discouraged in this population. Additionally, the risk of any complication (perforation or bleeding) is theoretically increased in other EDS subtypes, although not quantifiable and although no patient in our cohort underwent any complication. CT colonography could be an alternative option to rule out polyps or tumor but one should keep in mind the risk of repeated irradiating exams in this population with frequent orthopedic complications.

Our study has several limitations. The first one is the absence of control group (except regarding GIQLI for which we used a historical control French population) which precludes definitive conclusions from this survey. However, there is an abundant literature about FBD and GERD and comparisons with reported natural frequencies and severity in historical populations allow us to assume that gastrointestinal manifestations in EDS are particularly common and linked to the condition. For instance, a French survey conducted on a nationally representative sample reported a prevalence of IBS (according to the Rome II criteria which were slightly different from the Rome III criteria) to be lower than 5% [14]. Another epidemiologic mail survey found that prevalence of GERD reached 8% in another sample of 8000 subjects representative of the French adult population [15]. In addition, we cannot rule out the possibility of response bias, although our percentage of returned questionnaires was slightly higher than the generally required 60% response rate [16]. Although many patients of the questioned cohort had genetic testing, we were not allowed to collect these data in the present study, which is another potential deficiency. However, most of our population was affected by hypermobility type, consistently with literature and the genetic basis of this EDS form is lacking. At last, we cannot establish how many patients with differential diagnosis (such as neuromuscular disorders or other connective tissue disorders) were excluded from the present cohort.

In summary, our study is to date the largest conducted survey specifically assessing the natural frequency, nature and impact of functional gastrointestinal manifestations in EDS. It emerges that digestive manifestations are extremely common, most frequently nonspecific and not serious but with major consequences on quality of life. A systematic clinical assessment should be recommended in EDS population and further studies are needed to elucidate the pathophysiology of these disorders and to improve therapeutic management.

## Supporting Information

Appendix S1 Questionnaire.
(DOCX)
