# Supplementary material for: Functional Digestive Symptoms and Quality of Life in Patients with Ehlers-Danlos Syndromes: Results of a National Cohort Study on 134 Patients
Source: PLoS One. 2013 Nov 22;8(11):e80321. doi: 10.1371/journal.pone.0080321 (PMC3838387; doi:10.1371/journal.pone.0080321)
Supplement: Appendix S1 — Questionnaire. (DOCX) [file pone.0080321.s001.docx]

***Ehlers-Danlos syndrome***

First name (three first letters)

Last name (three first letters)

Date of birth

Gender

Weight

Height

Do you smoke tobacco?
If yes, how many cigarettes per day?

Do you sometimes drink alcohol?

If yes, how many glasses (mean) per week?

Date of EDS diagnosis

Date of EDS first symptoms

EDS subtype

Except for EDS, do you have any other major condition?

If yes, which one?

Do you work?

If no, since when did you have to interrupt your professional activity?

**GIQLI**

1 . During the last 15 days, you have had a stomach ache

always (0), most of the time (1), sometimes (2), rarely (3), never (4)

2 . During the last 15 days, you had the feeling of having bloated stomach

always (0), most of the time (1), sometimes (2), rarely (3), never (4)

3 . During the last 15 days, you had the feeling of having a lot of gas in the stomach

always (0), most of the time (1), sometimes (2), rarely (3), never (4)

4 . During the last 15 days, you 've been bothered the issue of "winds"

always (0), most of the time (1), sometimes (2), rarely (3), never (4)

5. During the last 15 days, you 've been bothered by belching or referrals

always (0), most of the time (1), sometimes (2), rarely (3), never (4)

6 . During the 15 days were you embarrassed by noises "gurgling" in the belly

always (0), most of the time (1), sometimes (2), rarely (3), never (4)

7 . During the last 15 days, you 've been bothered by frequent bowel movements

always (0), most of the time (1), sometimes (2), rarely (3), never (4)

8 . During the last 15 days, you ate with pleasure and appetite

9 . Because of your illness, you are required to remove certain foods

10 . During the last 15 days, you have been able to overcome daily problems

11 . During the last 15 days, how many times your illness made you sad?

always (0), most of the time (1), sometimes (2), rarely (3), never (4)

12 . During the last 15 days, how many times have you been anxious because of your illness?

always (0), most of the time (1), sometimes (2), rarely (3), never (4)

13. During the last 15 days, how many times have you felt joy of living

always (0), most of the time (1), sometimes (2), rarely (3), never (4)

14. During the last 15 days, how many times have you been frustrated because of your illness?

always (0), most of the time (1), sometimes (2), rarely (3), never (4)

15 . During the last 15 days, how often did you felt tired?

always (0), most of the time (1), sometimes (2), rarely (3), never (4)

16. During the last 15 days, how many times have you been painful?

always (0), most of the time (1), sometimes (2), rarely (3), never (4)

17. During the last week, did you awake during the night?

always (0), most of the time (1), sometimes (2), rarely (3), never (4)

18 . Since you are sick, have you been chagrined changes in your appearance?

For a very large part (0), for an important part (1), a little (2), a few (3), not at all (4)

19. To what extent is that it has reduced your requirement physics in general?

enormously (0), a lot (1), some (2), a little (3), not at all (4)

20 . Because of your health, you have lost your endurance?

For a very large part (0), for an important part (1), a little (2), a few (3), not at all (4)

21. By your illness you feel the loss of your tone?

major (0), moderate (1), small (2), insignificant (3), none, you feel well (4)

22. During the last 15 days, how many times have you been able to do your usual activities (work, school, cleaning, etc.) ?

never (0), rarely (1), sometimes (2), most of the time (3), always (4)

23. During the last 15 days, you have been able to attend your usual leisure or new activities

never (0), rarely (1), sometimes (2), most of the time (3), always (4)

24 . During the last 15 days, have you been bothered by medical treatment?

enormously (0), a lot (1), some (2), a little (3), not at all (4)

25 . To what extent your illness she disrupts your relationships with others (family or friends)?

For a very large part (0), for an important part (1), a little (2), a few (3), not at all (4)

26. To what extent has your illness harmed your sex life?

For a very large part (0), for an important part (1), a little (2), a few (3), not at all (4)

27. During the last 15 days, how many times have you been inconvenienced by liquid or food in the mouth (regurgitation)?

always (0), most of the time (1), sometimes (2), rarely (3), never (4)

28. During the last 15 days, have you felt forced to decrease the speed with which you eat?

always (0), most of the time (1), sometimes (2), rarely (3), never (4)

29. During the last 15 days, you had problems to swallow

always (0), most of the time (1), sometimes (2), rarely (3), never (4)

30 . During the last 15 days, you have felt the need urgent need to defecate

always (0), most of the time (1), sometimes (2), rarely (3), never (4)

31. During the last 15 days, you have been inconvenienced by diarrhea

always (0), most of the time (1), sometimes (2), rarely (3), never (4)

32. During the last 15 days, you have been inconvenienced by constipation

always (0), most of the time (1), sometimes (2), rarely (3), never (4)

33. During the last 15 days, you have been inconvenienced by nausea

always (0), most of the time (1), sometimes (2), rarely (3), never (4)

34. During the last 15 days, you were worried by presence of blood in stool

always (0), most of the time (1), sometimes (2), rarely (3), never (4)

35. During the last 15 days, you have been inconvenienced by burn or acidity back in the chest

always (0), most of the time (1), sometimes (2), rarely (3), never (4)

36. During the last 15 days, you have been inconvenienced by incontinence for stool?

always (0), most of the time (1), sometimes (2), rarely (3), never (4)

**GERD/dyspepsia**

Are you currently or have you had recently one or more of the following symptoms?

- Heartburn behind the sternum
- Acid regurgitations in the throat
- Abovementioned manifestations increased when lying down or bending forward
- Chronic cough
- Laryngitis (hoarseness)
- Erosion of dental enamel
- Asthma (pulmonary wheezing with shortness of breath)
- Dysphagia (sensation of food blockage in the path of the esophagus after swallowing)
- Stomach pain
- Nausea
- Sensation of full stomach
- Eructation/belching

Have you already had one (or more) upper gastrointestinal endoscopy?

If yes, what was the result?

For the abovementioned symptoms, have you ever had one (or more) of the following treatments (and if yes, quote from 0 to 5 their effectiveness):

- Proton pump inhibitor (PPI) like omeprazole, Mopral, Inexium, Inipomp, Eupantol, Pariet, Ogast, Lanzor
- Prokinetic like Primpéran, Motilium, Oropéridys, domperidone
- Antacid like Maalox
- Alginate like Gaviscon

Have you already had one (or more) upper colonoscopy?

If yes, what was the result?

For the abovementioned symptoms, have you ever had one (or more) of the following treatments (and if yes, quote from 0 to 5 their effectiveness):

- Macrogol (like Transipeg, Forlax, Movicol)
- Bulking agent (like Spagulax, Polykaraya)
- Stool softener (like Lansoÿl, paraffin)
- Enema (like Normacol)
- Suppository (like Eductyl)
- Stimulant laxatives (like Dulcolax, Contalax, Péristaltine, Bourdaine Boiron, laxatives herbal teas)

Have EDS GI symptoms started before EDS formal diagnosis?

Have EDS GI symptoms started before other EDS symptoms?

Do you have any other GI manifestation which was not mentioned in the present questionnaire?

Do you currently take (or have you been taking repeatedly) morphine treatments?

Have you ever asked (or used) for complementary alternative medicines to alleviate your GI symptoms?

If yes, which ones?

What was their effect?

**Functional Bowel Disorder (Rome III)**

Do you have recurrent abdominal pain or discomfort at least 3 days per month in the last 3 months?

If yes, do you have also:

- Improvement with defecation?
- Onset associated with a change in frequency stool?
- Onset associated with a change in form (appearance of stool)?

Do you have recurrent feeling of bloating or visible distention at least 3 days/month in 3 months?

If you feel having constipation, which propositions concern your bowel habits?

- Straining during at least 25% of defecations?
- Lumpy or hard stools in at least 25% of defecations?
- Sensation of incomplete evacuation for at least 25% of defecations?
- Sensation of anorectal obstruction/blockage for at least 25% of defecations?
- Manual maneuvers to facilitate at least 25% of defecations (eg, digital evacuation, support of the pelvic floor)?
- Fewer than 3 defecations per week?
- Loose stools are rarely present without the use of laxatives?

**Score KESS**

**1. Duration of constipation**

0-18 months 0

18 months to 5 years 1

5-10 years 2

10-20 years 3

>20 years (or all life) 4

**2. Laxative use**

None 0

Laxatives pm or for short duration 1

Laxatives regular, long duration 2

Laxatives long duration, ineffective 3

**3. Frequency of bowel movement**

**(using current therapy)**

1-2 times / 1-2 days 0

2 or less times / week 1

Less than once per week 2

Less than once per 2 weeks 3

**4. Unsuccessful evacuatory attempts**

Never / rarely 0

Occasionally 1

Usually 2

Always = manual evacuation 3

**5. Feeling incomplete evacuation**

Never 0

Rarely 1

Occasionally 2

Usually 3

Always 4

**6. Abdominal pain**

Never 0

Rarely 1

Occasionally 2

Usually 3

Always 4

**7. Bloating**

Never 0

Perceived by patient only 1

Visible to others 2

Severe causing satiety or nausea 3

Severe with vomiting 4

**8. Enemas / Dictation**

None 0

Enemata / suppositories occasionally 1

Enemata/suppositories regular 2

Manual evacuation occasionally 3

Manual evacuation always 4

**9. Time taken (minutes in lavatory/attempt)**

< 5 minutes 0

5-10 minutes 1

10-30 minutes 2

> 30 minutes 3

**10. Difficulty evacuating (causing a painful evacuation effort)**

Never 0

Rarely 1

Occasionally 2

Usually 3

Always 4

**11. Stool consistency (without laxatives)**

Soft / loose/normal 0

Occasionally hard 1

Always hard 2

Always hard, usually pellet-like 3
